# ATP1A3 Acts as a Potential Anti-oncogene in Glioblastoma *via* the Antagonizing Interaction with Small Nuclear Ribonucleoprotein Polypeptide G

**DOI:** 10.2174/011570159X361656250128073206

**Published:** 2025-03-11

**Authors:** Shuang Zou, Bing Qin, Qi Chen, Zhiwei Shen, Qichang Liu, Xiangdong Zhu, Yulong Lan

**Affiliations:** 1 Department of Neurosurgery and Neurology, Key Laboratory of Precise Treatment and Clinical Translational Research of Neurological Diseases, Second Affiliated Hospital, School of Medicine, Zhejiang University, Hangzhou, Zhejiang, China;; 2 Key Laboratory of Neuropharmacology and Translational Medicine of Zhejiang Province, School of Pharmaceutical Science, Zhejiang Chinese Medical University, Hangzhou, China;; 3 Interdisciplinary Institute for Medical Engineering, Fuzhou University, Fuzhou, China;; 4 Department of Internal Medicine, Yale University School of Medicine, New Haven, Connecticut, USA

**Keywords:** Sodium pump α3 subunit (ATP1A3), small nuclear ribonucleoprotein polypeptide G (SNRPG), glioblastoma, nanomedicine, therapy, antagonizing interaction

## Abstract

**Background:**

The sodium pump α3 subunit (ATP1A3) is associated with various brain's physiological and pathological mechanisms. However, its molecular mechanisms and cellular targets in glioblastoma (GBM) are poorly understood.

**Methods:**

Bioinformatics and phosphor-proteomics analysis, target fishing experiment, confocal immunofluorescence, molecular cloning, and western blot techniques were carried out to elucidate probable downstream signaling pathways. Then GBM xenografts were established to assess potential molecular mechanisms of ATP1A3 associated with its *in vivo* anti-glioma impacts.

**Results:**

The mechanistic analyses indicated that the antagonism between ATP1A3 and small nuclear ribonucleoprotein polypeptide G (SNRPG) could suppress GBM growth. ATP1A3 inhibits SNRPG-induced GBM epithelial-mesenchymal transition, and SNRPG decreases ATP1A3 by increasing phosphorylation at S643. As a negative feedback loop, ATP1A3 overexpression causes a reduction of SNRPG-induced invasion-metastasis cascades *via * regulating KLF9. Furthermore, by using artificial intelligence (AI) techniques, we have also exerted the design and application of a synthetic peptide (ATP1A3-S643 peptide), which could be the potential inhibitor of ATP1A3 phosphorylation. To better explore the anti-glioma effect of ATP1A3 activation, a bioengineering nanomedicine capable of on-demand ATP1A3 activator delivery to the brain for GBM has also been developed in this work, which exhibited an improved therapeutic efficacy in the ATP1A3-targeted treatment of glioma.

**Conclusion:**

ATP1A3 is a potential anti-glioma treatment target, and its activation critically depends on its antagonizing interaction with SNRPG.

## 1 INTRODUCTION

Glioma is the leading type of primary central nervous system tumor, comprising about 60-70% of primary brain tumors, and the most aggressive type is glioblastoma-IDH wildtype (GBM), with a survival rate of only a few months without treatment [1]. The global impact of GBM is likely to rise, partly due to the aging population and unfavorable prognosis. Conventional therapies for GBM, including surgical resection, chemotherapy, and radiotherapy, have not significantly improved survival rates [2]. Currently, new therapeutic targets and improved GBM prognosis are urgently required. Therefore, understanding enhanced GBM malignancy mechanisms could contribute to more efficacious treatment. Although various GBM biomarkers have been identified, their clinical implementation is difficult because of their heterogeneity.

Eukaryotic cell membranes have numerous sodium pumps (Na^+^/K^+^-ATPase). In humans, these pumps have four α (ATP1A1-1A4) and three β (ATP1B1-1B3) isoforms [3]. The sodium pump α3 subunit (ATP1A3) gene encodes the Na^+^/K^+^-ATPase a3 isozyme, primarily highly expressed in neurons and the brain. This isozyme acts as the catalytic domain of the pump as it comprises Na^+^, ATP, and K^+^ binding sites and cardiac glycosides (pump suppressors). The ATP1A3 is essential for the maintenance of electrochemical gradients in neuronal functions [4] as it is exclusively expressed in neurons, and others are not [5]. Its dysfunction has been determined in individuals with different diseases, for instance, alternating relapsing encephalopathy with cerebellar ataxia, hemiplegia of childhood, fever-induced paroxysmal weakness and encephalopathy, and rapid-onset dystonia-parkinsonism [6-8]. Recently, increased sodium pump expression on mammalian cell membranes was linked with the incidence, initiation, and cancer progression [4]. Our previous investigation has indicated the presence of ATP1A3 on the GBM cell membrane surface, indicating its potential as a therapeutic target [9]; however, further research is required. ATP1A3 exerts a potential impact on different cancers, such as medulloblastoma, hepatoma, and glioma [10-12]. Understanding its role in GBM can improve the diagnosis and treatment paradigm, thereby improving prognosis. However, the exact function of ATP1A3 in GBM remains undetermined. Intriguingly, for the first time, our previous research has already indicated that the α3 subunit in the cancer cell membrane can be a novel anti-glioma drug target [13, 14]. However, systematic research on its precise roles in GBM requires attention, and more efforts should explore its therapeutic effect in GBM and the related molecular pathways for GBM therapy.

Small nuclear ribonucleoprotein polypeptide G (SNRPG), or Smith protein G, is important for the synthesis of spliceosomal uridyl-rich small nuclear ribonucleoprotein (U snRNPs; U1, U2, U4, and U5), precursors of minor and major spliceosome [15]. The literature indicates that this splicing machinery component induces mRNA binding and processing and is critically linked with initiation and cancer progression [16]. SNRPG has become a research hotspot because of its role in development and tumorigenesis [17]. Interestingly, it has broad interaction networks, and its activities are primarily induced by protein-protein interactions (PPIs), making it an efficient anti-cancer treatment target in PPI-linked drug technology [15]. Previously, we elucidated the anticancer pathway of SNRPG inhibition in GBM and furnished significant evidence for its potential as a new drug candidate against GBM [18]. Identifying specific SNRPG inhibitors may establish a new model for detecting and advancing molecular-target-related GBM treatments. However, its exact role needs elucidation.

Achieving efficient brain drug delivery is the main challenge in developing therapeutics for CNS conditions. The primary challenge is the blood-brain barrier (BBB), which blocks the entry of most molecules in the bloodstream, particularly large-molecule drugs and nanomedicines [19]. Thus far, the development and application of multifunctional nanosystems employing nanomaterials for tumor theranostics have garnered extensive attention in the clinic. It is known that under normal physiological conditions, the presence of tight junctions (TJ) *etc*., between cerebral microvascular endothelial cells maintains the closure of the BBB. The integrins (like IFA-1 or VLA-4) in microglial extracellular vesicles (EVs) could bind to cell adhesion molecules (like ICAM-1 or VCAM251) in cerebral endothelial cells, which trigger the intracellular signaling cascade and induce the damage of TJ and formation of a gap at BBB, finally leading to the probability of penetrating BBB [20]. Besides, microglial EVs could be recruited by multiple cytokines in the GBM microenvironment, leading to their enrichment in GBM tissues [20]. In our current research, we have proposed an innovative strategy in which an EV-engineered nanomedicine was employed to enhance the therapeutic efficacy of the ATP1A3 activator. The findings from the *in vivo* study indicated that injecting this bio-engineered agent systemically not only offered effective protection against GBM but also greatly reduced tumor growth and extended the survival rate.

Currently, the anti-cancer effect of ATP1A3, its association with SNRPG in human cancers, and their interactions are undetermined. This investigation studies the interaction of ATP1A3 with SNRPG in GBM cells to highlight the activity of ATP1A3 in regulating the invasion-metastasis cascade of GBM. Furthermore, we have also assessed the anti-glioma effects of the synthetic peptide designed for inhibiting ATP1A3 Ser643 phosphorylation. Intriguingly, by using artificial intelligence (AI) techniques, we have also exerted the design and application of synthetic peptides (ATP1A3-S643 peptide), which could be the potential inhibitor of ATP1A3 phosphorylation. Besides, a bioengineering nanomedicine capable of on-demand ATP1A3 activator delivery to the brain for GBM has also been developed in this work, which exhibited an improved therapeutic efficacy in the ATP1A3-targeted treatment of GBM. All these investigations can help clarify the treatment effect of ATP1A3 activation during GBM treatment.

## 2 MATERIALS AND METHODS

### 2.1 Antibodies and Reagents

GAPDH, β-actin, ATP1A3 FN1, Ki-67, Snail, SNRPG, and Vimentin primary antibodies, as well as the matching secondary antibodies, were provided by Saituo Biotech (Dalian, China). Trypsin, FBS, and DMEM were supplied by HyClone Laboratories (USA). Abcam (UK) provided the human recombinant ATP1A3 protein (ab152203), while Sigma (USA) supplied protease inhibitors and PBS. The synthetic peptide (ATP1A3-S643 peptide) was designed by Target Molecule Company (Shanghai, China) and synthesized by Genscript Company (Hangzhou, China). All additional chemicals, unless otherwise stated, were from Sigma.

### 2.2 Bioinformatic Analysis Regarding Various Na+/K+-ATPase Subunits Expressions in GBM Patients

Bioinformatic analysis of the association between different GBM subtypes and mRNA levels of all Na^+^/K^+^-ATPase subunits in GBM patients has been exerted. According to Fig. (**1A**): (1), multi-omics profiles of 366 GBM patients were acquired [21]. The clinicopathological and survival data were retrieved from cBioPortal (https://www.cbioportal.org/). The expression profiles of 143 primary tumors were imported from UCSC Xena (https://xenabrowser.net/datapages/) along with 5 adjacent normal tissue samples. To ensure comparability among samples, the values of fragments per kilobase million (FPKM) were converted into transcripts per kilobase million (TPM) [22]. (2), Furthermore, four GBM subtypes were identified by retrieving 840 genes from the literature and selecting gene markers for each molecular subtype based on transcriptome expression data, including mesenchymal, proneural, neural, and classical [23]. Each sample was classified as one of the four subtypes using the nearest template prediction with these markers [24]. (3) Lastly, hierarchical clustering was performed for elucidating transcription expression data *via* a Euclidean distance measurement with Ward’s clustering method using the R package ClassDiscovery. In Fig. (**1B**), the mRNA levels of all Na^+^/K^+^-ATPase subunits in different GBM subtypes were assessed from the TCGA-GBM data *via* the cBioPortal online tools (http://www.cbioportal.org/). The heatmaps of the mRNA expressions of Na^+^/K^+^-ATPase subunits in different subtypes of GBM were generated by online tools of the cBioPortal browser.

### 2.3 Cells

Cell lines were validated through short tandem repeat (STR) profiling (GeneChem Co., Ltd., Shanghai, China). Mycoplasma contamination was excluded using the Mycoplasma Detection Test (Kit-Quick, Biotool, Shanghai, China), and U87-MG cells (American Type Culture Collection, Manassas, VA, United States) were cultivated in DMEM with 10% FBS at 37°C and 5% CO_2_.

### 2.4 Viability Assays

Viability was examined with CCK-8 assays. Cells (5 × 10³/well) were grown overnight in 96-well plates, followed by the replacement of the media with fresh media containing ATP1A3-S643 peptide (5 μM in < 0.1% DMSO) and culture for 48 h when CCK-8 was added and absorbances were read at 450 nm using an EnSpire^®^ Multimode Plate Reader (Perkin Elmer, USA). The viability of the vehicle control represented 100%. Triplicate tests were performed.

### 2.5 Apoptosis Assays

Apoptosis was examined using an Annexin V-FITC kit (USA). Cells were grown in 6 cm culture dishes until they reached maximum confluence. Following 12 h of treatment, cells underwent three washes and digestion with trypsin with no EDTA. After resuspension in BSA (0.2%) and centrifugation (600 g, 5 min), the pellet was reconstituted in binding buffer (500 µL). Following treatment with Annexin V-FITC (5 µL) and PI (5 µL) for 5-15 min at ambient temperature away from light, proportions of apoptotic cells were assessed on the FACS system (FACS Accuri C6, CA, USA).

### 2.6 Target Fishing

All the protocols in the present study followed the Plexera rules and regulations [25, 26]. The PlexArray^®^ HT and Molecular Chip (Plexera LLC, USA) was utilized for target fishing. (A) Four Fixed Small Molecular Chips were utilized, two of which were immobilized with ATP1A3 and two with methanol (blank control). Two chips were incubated with a solution of recombinant protein (0.31% glutathione + 0.79% Tris HCl, pH: 8.00) used as a negative control and two (positive control) with ATP1A3. (B) U87-MG cells were lysed, diluted to 1 mg/mL, and separated into positive and negative controls. (C) For target fishing, the SPR protocol was in two: (1) Positive control: U87-MG cell suspensions were applied to the ATP1A3 chip mounted on the SPR device, capturing target proteins within the lysate. (2) Blank control: The chip was immersed in recombinant protein solution before performing the above steps. (D) Target protein retrieval using glycine-HCl: After elution from the chip, the target proteins were digested with trypsin and then identified by HPLC-MS/MS. The experimental parameters are provided in Table **S1**.

### 2.7 Western Blot Test

The extracted proteins were quantified using a single kit (Beyotime Biotechnology, China). Following denaturation, 50 μg of proteins were separated on 8-12% SDS-PAGE and electroblotted to PVDF membranes (Millipore, USA) labeled with the indicated antibodies and observed using enhanced chemiluminescence. GAPDH and β-actin represented the loading controls.

### 2.8 ATP1A3 Overexpression by Transfecting Lentiviral Vectors

YouBio (Changsha, Hunan, China) provided lentiviral vectors. pCDH-GFP-PURO-3xFlag vector was ligated with ATP1A3 cDNA sequence after cloning to generate lentiviruses 245, 246. 293T cells were used for plasmid insertion. After 48 h, supernatants were removed, followed by filtration (0.45 μm). For viral infection, cells were mixed with the virus medium (1:5) for 24 h. Stably overexpressing ATP1A3 lines were selected with 2 μg/mL puromycin (Sigma).

 The primers used for ATP1A3 were as follows: Forward, 5'-AGGTCGACTCTAGAGGATCCCGCCACCATGGGG-GACAAGAAAGATGACAAGGAC-3'; Reverse, 5'-TCCT-TGTAGTCCATACCGTAGTAGGTTTCCTTCTCCACCC-AACC-3'. The cells that overexpressed ATP1A3 were termed lenti ATP1A3, while controls were described as lentivectors.

### 2.9 ATP1A3 Silencing using Short Hairpin RNAs (shRNAs)

Synbio Tech (Suzhou, Jiangsu, China) provided the lentiviral vectors. Negative control shRNA and ATP1A3-shRNA were cloned into the PLKO.1 vector. For lentivirus generation, plasmids encoding Rev, Gag, and VSVG were inserted into 293T cells. Viral supernatants were harvested after 48 h, filtered (0.45 μm), and used for infections by mixing with cancer cells (5:1 ratio) for 24 h. Cell lines exhibiting stable ATP1A3 knockdown were chosen by administering approximately 2 μg/mL puromycin, and the surviving cells were employed for further studies. The shRNA sequences with the highest efficiency in targeting ATP1A3 were: (F: 5'-CCGGCAAACCTCTCTCCTCTCTCTTCTCGAGAAG-AGAGAGGAGAGAGGTTTGTTTTTG-3'; R: 5'-AATTC-AAAAACAAACCTCTCTCCTCTCTCTTCTCGAGAAG-AGAGAGGAGAGAGGTTTG-3') [13]. The negative control was a non-silencing shRNA (5'-GCCTAACTGTGT-CAGAAGGAA-3').

### 2.10 Multiplexed Immunofluorescence Image Analysis

A TissueFAXS Spectra cytometer and StrataQuest software (TissueGnostics) were employed for the visualization and quantification of different fluorophores. Multi-spectral whole slide image was acquired by 20× objective lens. The fluorophores spectral library was established using individually stained sections to minimize the cross-fluorescence interference and the spectral unmixing algorithm. The images were acquired by Contextual Tissue Cytometry Image Analysis. For quantitation and phenotype analyses, the DAPI image was subjected to a nuclear segmentation algorithm to outline and identify each cell. All the markers’ locations and expressions were assessed in each cell. Lastly, corresponding algorithms based on different analysis requirements were developed and unified, and then each channel’s threshold was applied to all glioma samples to standardize the fluorescence and expression levels of each marker.

### 2.11 Confocal Immunofluorescence

Samples were fixed (paraformaldehyde, 30 min) before permeabilization with Triton X-100 (2‒5 min), blocking (1% BSA, 2% glycerol in PBS), and overnight treatment with anti-ATP1A3 or SNRPG antibodies in PBS with 1% BSA at 4°C. The samples were rinsed (PBS) and treated with secondary FITC-labeled antibodies (1 h, ambient temperature) before counterstaining with DAPI (Beyotime) and assessment and imaging under confocal microscopy (Leica DM 14000B).

### 2.12 Immunofluorescence Staining

Brain samples were preserved for 2 days in 4% PFA, followed by a progressive sucrose infiltration (15-30%) over the next 2 days. The tissues were then sectioned into 1.6 mm thick slices before washing in PBS (three times) and blocking (5% BSA, 1 h). After treatment with the primary antibody for a whole night at 4°C, the samples were treated (1 h) with Alexa Fluor-488 or -594 secondary antibodies (Invitrogen, USA), followed by counterstaining with DAPI and evaluation and imaging under light and confocal microscopy (Leica TCS SP5).

### 2.13 Co-immunoprecipitation (Co-IP)

Cell lysates (400 μg total protein) were treated with protein A/G-agarose beads (30 µg) and normal rabbit IgG (1 μg, Santa Cruz Biotechnology, USA) by gentle agitation at 4°C for 1 hour. After centrifugation, the supernatants were treated with anti-mouse ATP1A3 for 5-6 h at 4°C. After 3 rinses with chilled PBS with proteinase inhibitors, the immune complexes were removed using A/G agarose beads. After boiling the immune complexes in 2× loading buffer for 5 min, they were eluted and analyzed by Western blotting using rabbit anti-SNRPG and mouse anti-ATP1A3 antibodies.

### 2.14 Reporter Analysis

U87-MG cells (1.0 × 10^5^/well) were grown in 24-well plates for 24 h, transiently co-transfected for 16 hours with the reporter construct pGL3-SNRPG-pro-Wt or the pGL3-basic (1.5 mg/well) and KLF9 plasmid (0-3.0 mg/well). Dual-luciferase reporter assays (Promega) were utilized according to directions for measuring the luciferase activity. Renilla luciferase reporter plasmid was utilized as endogenous control and for normalizing Firefly luciferase values. The results of three separate assays are shown as mean ± SD.

### 2.15 Chromatin Immunoprecipitation Assay (ChIP)

Per the manufacturer's guide, the ChIP analysis was performed *via* Enzymatic Chromatin IP Kit (SimpleCHIP Plus, Cell Signaling Technology). For KLF9, IgG and H3 were used as negative and positive control antibodies, respectively. Quantitative Real-time PCR reaction and DNA agarose gel quantified the target DNA fragment. Seven sets of primers covering SNRPG promoters from about -200 kb to 1000 were prepared, and their relative average ChIP intensities were measured. The sequence of primer pairs was: Forward, 5’- AGACCATCCTGGCTAACACG -3’; Reverse, 5’- CGATCTCGGCTCACTGCA -3’.

### 2.16 Site-directed Mutation for ATP1A3 (Ser643Ala)

A single-step PCR protocol performed site-directed ATP1A3 mutagenesis. Ser643Ala mutation was incorporated in pIRES2-EGFP-TRPM2 with the help of appropriate primer pairs by a Site-Directed Mutagenesis Kit (QuikChange, Agilent). Targeted site sequencing was used to confirm the mutant plasmid. ATP1A3 mutagenesis primers utilized were: Forward, 5’-TCAACATTCCCGTCGCCCAGGTTAACC-CC -3’; Reverse, 5’-GGGGTTAACCTGGGCGACGGG- AATGTTGA -3’. The recombinant plasmid with the ATP1A3 gene was utilized as the template. The following were the PCR parameters: 1 cycle, 94°C, 5 min; 35 cycles, 94°C, 30 sec; 58°C, 1 min, 72°C, 1 min; 72°C, 10 min. The template was amplified with Taq Plus DNA polymerase and processed by the Dpn1 restriction enzyme. The mutant sequences were verified by sequencing by GeneChem.

### 2.17 Quantitative Phosphor-proteome Analysis

This analysis was performed by PTM-Biolabs (Hangzhou) Co., Ltd. The analysis has three steps:

(1) Protein extraction: in liquid nitrogen, the sample was converted into powder, shifted in a 5-mL tube, mixed with four lysis buffer volumes (8 M urea and a 1% protease inhibitor mixture), and sonicated thrice with a Scientz processor. For PTM assays, suppressors were mixed with lysis buffer, *i.e*., 1% phosphatase inhibitor for (phosphorylation and 3μM TSA + 50 mM NAM for acetylation. After centrifugation (12000 g, 10 min, 4°C) for removal of debris, the protein contents of the supernatants were determined by BCA assays.

(2) Digestion with trypsin: proteins were reduced (5 mM dithiothreitol, 30 min, 56°C) and alkylated (11 mN iodoacetamide, 15 min) in darkness at environmental temperature, diluted (100 mM TEAB, <2 M urea), and digested twice, first with 1:50 trypsin: protein ratio overnight and then with a 1:100 ratio for 4 h. Lastly, the C18 SPE column was used for peptide desalting.

(3) PTM enrichment using biomaterials: initially, the peptide solutions were mixed well with a suspension of IMAC microspheres in a loading buffer (50% acetonitrile, 0.5% acetic acid). To prevent non-specific adsorption of peptides, the IMAC microspheres were washed twice, first with 50% acetonitrile/0.5% acetic acid and then with 30% acetonitrile/0.1% acetic acid. Phosphopeptides elution was performed with buffer including 10% NH_4_OH with vibration. The phosphopeptide supernatant was lyophilized for LC-MS/ MS and subsequent bioinformatics analyses.

### 2.18 Molecular Modeling

The Uniprot database (https://www.uniprot.org/) was utilized for acquiring PDB codes of ATP1A3 and SNRPG proteins.

(1) Site mutations or modifications: The Pymol software (version 2.5) was used to mutate the amino acid residue at ATP1A3 Ser643 (Ser to Ala). Vienna-PTM software (version 2.0) for phosphorylation modification of ATP1A3 protein at ATP1A3 Ser643.

(2) Molecular dynamics simulation of the modified or mutated ATP1A3 protein structure was exerted using Gromacs software (2022.5 released) (Simulation process: Energy minimization-NVT canonical ensemble-NPT isothermal, isobaric ensemble).

(3) With the help of ClusPro software (version 2.0), molecular docking between the modified or mutated ATP1A3 was performed with the SNRPG protein structure. The docking poses a PDB structure file, and the docking binding energy was obtained to quantify the binding affinity.

### 2.19 Immunohistochemistry (IHC) and Microarray

The Outdo Biotech (Shanghai, China) provided human tissue microarrays with 153 glioma specimens (Table **S2**). Briefly, the tissue samples were deparaffinized, then rehydrated and subjected to antigen retrieval in a solution with a pH of 6.0, followed by three rounds of microwave heating at medium power (10 min each). After blocking (3% BSA) and primary anti-ATP1A3 treatment (1:10), treatment with a secondary HRP-conjugated anti-rabbit IgG antibody, the slides were stained with hematoxylin. Ordinary rabbit IgG was the negative control instead of the primary antibody. An Olympus microscope was used for counting positive cells in 3-4 fields.

### 2.20 Design of Protein-binding Peptides that Could Bind to ATP1A3 Ser643

Designing new peptides using deep-learning methods has been performed according to a general deep-learning framework described by David Baker and colleagues [27]. We generate the binder scaffold using the Binder Design module of RFdiffusion, which is indicated in Cyan [27]. Then we design binder sequences with ProteinMPNN, and validate and rank the folding ability, as well as the ATP1A3 binding conformation of designed sequences using Alphafold Multimer. Finally, we verified the binding stability by performing a molecular dynamics simulation of the candidate sequences, and we finally obtained 10 short sequence structures of peptides stably binding to the ATP1A3 S643 phosphorylation site. Among all these peptides, the most prominent one could be the highest PPI Affinity score and the lowest pAE score.

### 2.21 Synthesis of EV-DSPE-SS-ATP1A3-S643

(1) Synthesis of the DSPE-SS-COOH. 100 mg DSPE was dissolved in 5 ml of Trichloromethane, then 2 ml dimethylformamide including dithiodipropionic acid (3.0 eq.), PyBOP (2.0 eq.) and DIPEA (3.0 eq.) was added. The reaction was performed at room temperature for 0.5 h. Then, the reaction solution was concentrated under reduced pressure and precipitated under a large amount of cold water, and then the products were collected by filter extraction. After washing with pure water 3 times and drying under vacuum, DSPE-SS-COOH could be obtained.

(2) Synthesis of the DSPE-SS-CS6. 50 mg DSPE was dissolved in 3 ml of Trichloromethane, then 2 ml dimethylformamide including CS6 (1.1 eq.), DMAP (0.5 eq.), HOBt (0.5 eq.) and DIC (0.5 eq.) was added. The reaction was performed at 50^o^C for 12 h. Then, the reaction solution is concentrated under reduced pressure and purified by column chromatography. After drying under vacuum, DSPE-SS-CS6 could be obtained.

(3) Fabrication of EV-DSPE-SS-ATP1A3-S643: The EV of the microglia cells and DSPE-SS-CS6 were separately dissolved into equal volume solutions and mixed (the ratio of EV albumen quality and DSPE-SS-CS6 was 1:1). The mixture was extruded 11 times with a 200 nm polycarbonate membrane using a liposome extrudator, then the EV-DSPE-SS-ATP1A3-S643 could be obtained.

### 2.22 Rodent Experiments

Ten microliters of cells (1 × 10^8^) were stereotactically injected into the brain of female BALB/c nude mice (adult 5-6 weeks old) (GemPharmatech Co., Nanjing, Jiangsu, China), and the animals were randomly categorized into 3 groups (the randomisation sequence was generated by using a random number generator). Following 10 days, the groups were subdivided and treated with the synthetic peptide (SP) at varying concentrations (0, 5, and 20 mg/kg vehicle). Only healthy animals were included for further experiments, and sick ones were excluded. Three mice in each group and nine mice in total were used in this experiment. To assess SP treatment, SP with different concentrations was given intraperitoneally twice weekly. Subsequently, glioma size was evaluated by euthanizing the animals with CO_2_ at the onset of symptoms. After that, the tumors were removed, after size evaluations by a ruler, formalin-preserved for histological examination, or frozen in liquid nitrogen for further experiments. IHC was used to determine the expressions of p-ATP1A3 Ser643, ATP1A3, Ki-67, and SNRPG. Briefly, tissue slices (4 μm) were incubated with ATP1A3 (1:500), p-ATP1A3 Ser643 (1:500), Ki-67 (1:1000), and SNRPG (1:500) antibodies. After that, the images were observed with a microscope (Leica DM 4000B).

In the second animal study, cell implantation was exerted in the same way as in the first animal study. The U87-MG cells were administered to female BALB/c nude mice (adult 5-6 weeks old) (GemPharmatech Co., Nanjing, Jiangsu, China), and then all animals were categorized into five groups randomly (the randomisation sequence was generated by using a random number generator). Only healthy animals were included for further experiments, and sick ones were excluded. Three mice in each group and nine mice in total were used in this experiment. Three mice in each group and fifteen mice in total were used in this experiment. Animals were observed and weighed every four days after cell implantation. After 10 days, the animals in each group were subdivided into subgroups and assigned to receive different treatments. The mice with tumors were divided into five groups randomly for treatment with (1) PBS, (2) EV, (3) ATP1A3-S643, (4) DSPE-SS-ATP1A3-S643, and (5) EV-DSPE-SS-ATP1A3-S643, respectively. All five groups of animals received intraperitoneal drug injections (twice each week). Bioluminescence imaging was performed 16 and 24 days after cell implantation, and one MRI coronal scanning was performed 32 days after cell implantation. Glioma sizes were evaluated by euthanizing the animals with CO_2_ at the onset of symptoms. The tumors were then removed for subsequent protein expression analysis.

All experiments complied with the NIH Guide (Bethesda, MD, USA) for the Care and Use of Laboratory Animals. Zhejiang University's Experimental Animal Center supplied all the animals (Certificate of Conformity: No. SCXK (Zhe) 2018-0006). Only the personnel authorized to keep laboratory animals and conduct animal experiments, who have not been involved in this study, were aware of the group allocation at the different stages of the experiment (during the allocation, the conduct of the experiment, the outcome assessment, and the data analysis). The mice were allowed to acclimatize for 2 weeks prior to experiments in a controlled environment with unlimited food/water access at 23°C, 50% humidity, and a 12/12-h light/dark cycle. Maybe some other potential confounders, such as the order of treatments and measurements or animal/cage location, were not controlled. Besides, the current study did not have humane endpoints. Throughout the experiment, every attempt was made to reduce suffering, and no animals were lost.

### 2.23 Statistical Analysis

Data analysis was conducted using GraphPad Prism 6.0. Between-group comparisons were performed with two-tailed Student’s and Welch’s t-tests. For multiple-group comparisons, two-way analysis of variance (ANOVA) followed by Bonferroni’s post-hoc test or one-way ANOVA followed by Tukey’s test were used. In most *in vitro* and *in vivo* experiments, Student’s t-tests were utilized for the determination of the *p*-values. Data are expressed as the mean ± SEM. *P*-values less than 0.05 were considered statistically significant, with the following significant levels: *p < 0.05, **p < 0.01, ***p < 0.001, and ****p < 0.0001.

## 3 RESULTS

### 3.1 The Outstanding Downstream Target of ATP1A3 in GBM

**Fig. (1):**
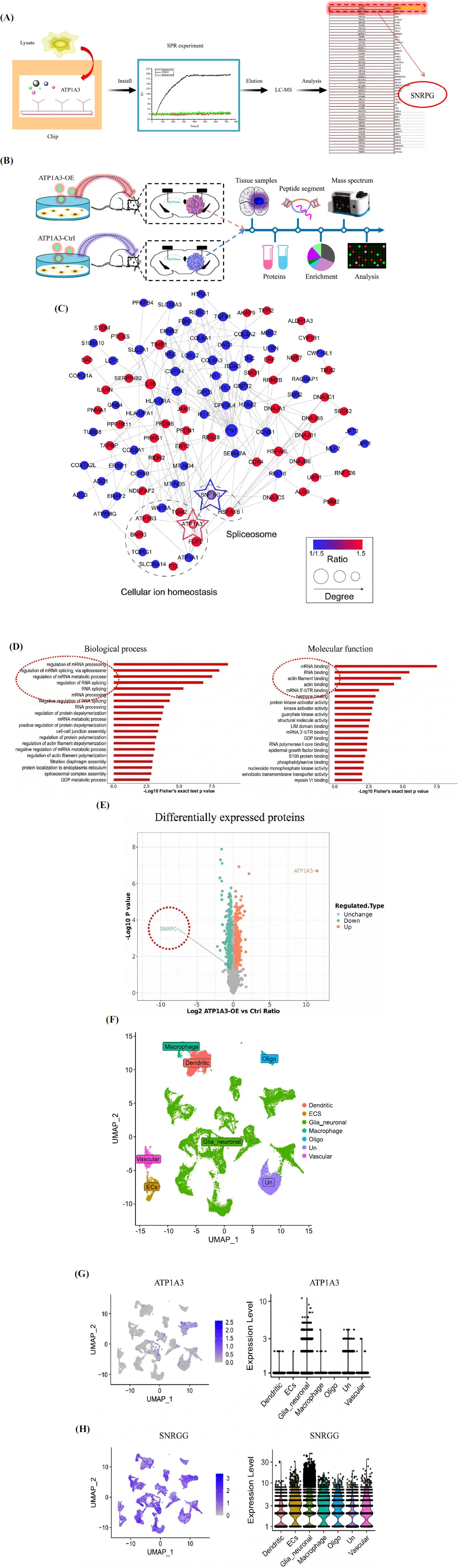
Identification of the downstream target of ATP1A3 protein. (**A**) A target fishing assay was carried out to determine primary ATP1A3 targeted proteins. Subsequent to the SPR assay, target proteins were eluted from the chip, trypsinized, and identified by HPLC-MS/MS. Quantitative MS analyses revealed that SNRPG was located in the ATP1A3 complexes. (**B**) Quantitative proteomics and phosphoromics analysis were used to analyze the downstream factors of ATP1A3 in GBM cells. (**C**) The differential proteins were extracted and aligned to the string database in the PPI-network analysis. Then proteins with > 0.4 confidence score (medium confidence) were selected for PPI analysis and visualization. The point's color reflects the protein's ratio value, and the point's size reflects the degree. This investigation focuses on ATP1A3 and SNRPG and highlights their involved mechanisms. (**D**) Gene Ontology (GO) enrichment analysis of modulated protein phosphorylation on the ontology of biological processes and molecular functions. (**E**) The volcano map further confirmed the downstream SNRPG protein expression. (**F-H**) Determining the distribution of *ATP1A3* in various cell subpopulations *via* single-cell RNA-Seq data analysis. (**F**) GBM cell subpopulations: Glia-neuronal, glia/neuronal cells; ECs, Endothelial cells; Oligo: oligodendrocytes; Vascular, vascular mural cells. (**G**) *ATP1A3* expression and box plots of the *ATP1A3* expression in various GBM subpopulations. (**H**) Expression of *SNRPG* and box plots of the *SNRPG* expression in various GBM subpopulations.

Our previous research has already confirmed the ATP1A3 expression status and prognostic value in glioma [13], indicating that ATP1A3 expression levels could be applied as a prognostic marker in glioma (Fig. **S1**). The potential downstream factors of ATP1A3 in GBM cells were identified with the help of the target fishing experiment (Fig. **1A**). Furthermore, HPLC-MS/MS identified 149 target proteins (Table **S3**). Moreover, SNRPG was identified among the target proteins extracted by ATP1A3 in U87-MG cells. Therefore, it was hypothesized that SNRPG might be associated with ATP1A3s’ anti-cancer effects in GBM. GBM xenografts were established to assess potential ATP1A3 downstream targets *in vivo*. The transplantable tumors were separated into ATP1A3-OE and control groups. Each group was labeled with TMT, and proteomics/phosphorylation affinity enrichment assay was performed, followed by HR-LC-MS/MS to measure the alterations in the whole phospho-proteome of glioma tissues (Fig. **1B**). Then the differential genes between these two groups were carried out, and the PPI network was analyzed following quantitative proteomics analysis (Fig. **1C**). Furthermore, quantitative phosphoromics revealed that increased ATP1A3 expression causes mRNA binding and induces processing pathways (Fig. **1D**). Since SNRPG could be an important mRNA processing component, current data validates the ATP1A3-induced SNRPG-related signaling. The volcano map further confirmed the downstream SNRPG protein expression (Fig. **1E**). Moreover, from the GEO database, single-cell RNA-Seq (scRNA-Seq) data of ATP1A3 and SNRPG in 12 GBM patients were assessed, and an expression matrix of 34,018 cells was observed for 19,043 genes through quality control. The distribution of these cells was visualized according to classical marker genes using UMAP (Fig. **1F**). UMAP visualization of the *ATP1A3* and SNRPG genes revealed them as well characterized in the corresponding cells (Fig. **1F**). Examples include the expression of *ATP1A3* and *SNRPG* in glia/ neuronal cells (Figs. **1G**, **1H**). These genes in different cell subpopulations were also plotted in expression box plots; it was revealed that *SNRPG* and *ATP1A3* were jointly specifically upregulated in glia/neuronal cells (Figs. **1G**, **1H**). Since the multifactorial connectivity of neurobiology to cancer malignancy, the so-called “cancer neuroscience” has become a research hotspot currently. These results could further convince us of their combined effect on the malignant proliferation of human glioma.

### 3.2 ATP1A3 Expression and its Relationship with SNRPG in Human GBM

**Fig. (2):**
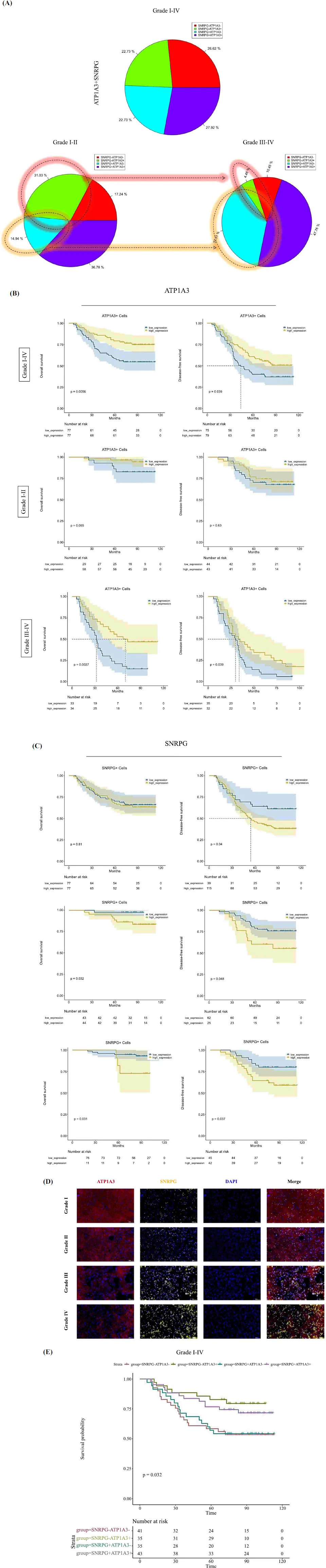
The correlation between ATP1A3 and SNRPG in glioma. (**A**) Percentage distribution of samples with different ATP1A3 and SNRPG expressions. (**B**) OS and DFS assessment of individuals with different ATP1A3 levels in glioma cells. (**C**) OS and DFS assessment of individuals with different SNRPG expressions in glioma cells. The log-rank test elucidated intergroup differences. Data are depicted as mean ± SEM. (**D**) Representative images of human glioma tissues using multiplexed immunofluorescence staining. Scale bar, 50 μm. (**E**) Kaplan-Meier analysis of OS in subgroups with high and low ATP1A3 and SNRPG expressions.

With multiplexed immunofluorescence staining, the potential association of ATP1A3 with SNRPG in 154 glioma tissues was analyzed. The clinical and pathologic characteristics are present in Table **S4**. Their expressions were analyzed in all grades of gliomas (Fig. **2A**), which revealed that high ATP1A3 expression was accompanied by low SNRPG expression and *vice versa*. In low-grade gliomas (grade I-II), those with high ATP1A3 and low SNRPG expressions had the most significant proportion (ATP1A3 high + SNRPG low, 31.03%), while gliomas with low ATP1A3 and high SNRPG expressions accounted for the least proportion (ATP1A3 low + SNRPG high, 14.94%). However, an opposite trend was observed in high-grade gliomas (grade III-IV); the proportion of gliomas with high ATP1A3 and low SNRPG expression markedly decreased (ATP1A3 high + SNRPG low, 4.48%), whereas those with low ATP1A3 and high SNRPG expression notably increased (ATP1A3 low + SNRPG high, 37.31%). Indicating the opposite expression trend of ATP1A3 and SNRPG protein and their co-expression patterns in different grades of gliomas (Fig. **2A**). Additionally, the clinical importance of ATP1A3 and SNRPG expressions in gliomas was determined, and it was revealed that an increased percentage of ATP1A3 cells in glioma patients was linked with prolonged OS and DFS than those with a reduced number of ATP1A3 cells (Fig. **2B**), whereas glioma patients with an elevated number of SNRPG cells had shorter OS and DFS than those with a reduced number SNRPG cells (Fig. **2C**). Furthermore, multiplexed immunofluorescence staining revealed that ATP1A3 and SNRPG proteins had opposite expression level trends in different glioma grades (Fig. **2D**). The prognostic value of ATP1A3 and SNRPG was assessed (Fig. **2E**), and a strong correlation was observed (*P* = 0.032). Specifically, the prognosis of patients with high ATP1A3 expression glioma was significantly better than low ATP1A3 expression patients, where glioma patients with increased ATP1A3 and reduced SNRPG expressions had the best prognosis (pale green color), while those with high ATP1A3 and SNRPG expressions had a relatively poorer prognosis (purple color). Thus, it was clear that the expression of ATP1A3 and SNRPG proteins jointly affects the prognosis of glioma patients. Overall, metastatically, ATP1A3 was negatively linked with SNRPG; increased ATP1A3 levels showed better glioma prognosis independent of SNRPG levels, whereas SNRPG-related prognosis depended on ATP1A3 levels.

### 3.3 ATP1A3 Inhibits SNRPG-induced GBM Epithelial-mesenchymal Transition (EMT) via Regulating KLF9 Promoter

**Fig. (3):**
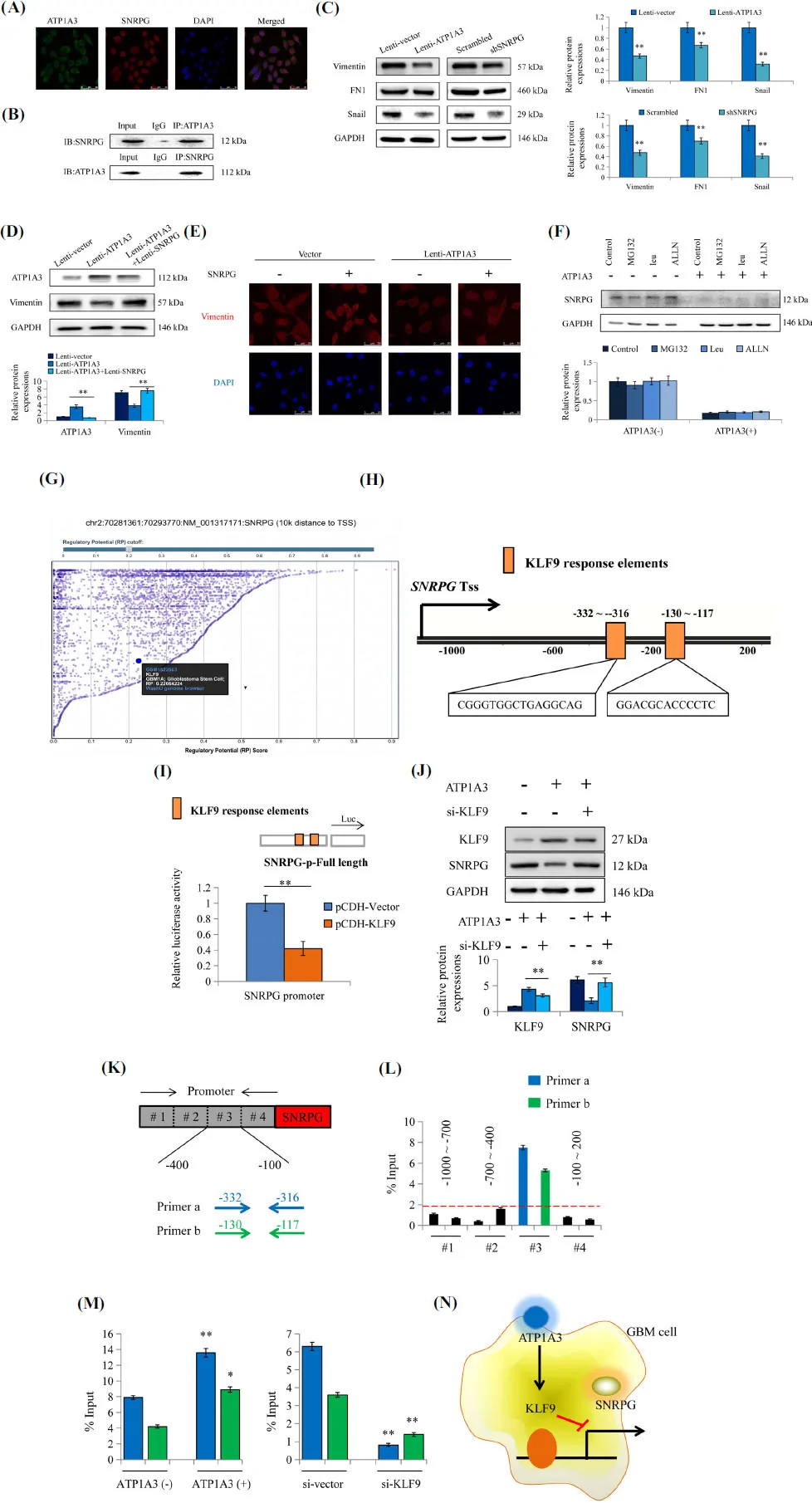
The influence of ATP1A3 on SNRPG-induced GBM EMT. (**A**) Confocal immunofluorescence analysis examined the localization of ATP1A3 and SNRPG proteins in the U87-MG cell line. (**B**) The CO-IP technique confirmed the direct interaction of ATP1A3 and SNRPG. (**C**) The expressions of EMT-related genes, including vimentin, FN1, and Snail, were validated by western blots with overexpressed ATP1A3-induced SNRPG knockdown. (**D**) The effect of ATP1A3 overexpression on EMT-related gene ‘vimentin’ was assessed *via* immunoblotting; furthermore, whether SNRPG overexpression reverses this ATP1A3-induced EMT change was also observed. (**E**) Immunofluorescence of Vimentin in ATP1A3 overexpressing U87-MG and corresponding control cells inoculated with or without SNRPG (5 ng/mL) was performed. DAPI was utilized for counterstaining nuclei. (**F**) U87-MG cells [with and without ATP1A3 (5 ng/mL)] were treated with various suppressors of protein degradation, such as leupeptin, MG-132, and ALLN (MG-101), and the expression of SNRPG was then assessed. (**G**) With Cistrome Database online tool, the potential SNRPG transcription factor was determined. (**H**) JASPAR Database (http://jaspar.genereg.net/) predicted potential KLF9 binding sites and their corresponding sequences in the SNRPG promoter region. (**I**) SNRPG promoter region fluorescence reporter vector containing the binding sites of KLF9 was transfected into GBM cells. Moreover, co-transfection of pCDH-Vector or pCDH-KLF9 was performed, followed by their fluorescence activity analysis after 48h. Student’s t-test, *p < 0.05; **p < 0.01. (**J**) Immunoblotting assay elucidated the impact of KLF9 knockdown on ATP1A3-mediated SNRPG inhibition. (**K**) Schematic outlining the SNRPG promoter region. Four primer sets comprising 8 primers covering SNRPG promoter; “a” and “b” indicates two primers covering -1000 to 200 bp region. The amplified fragments’ start and end are depicted. (**L**) Using ChIP and then RT-qPCR, the KLF9 binding sites in SNRPG were detected. (**M**) RT-qPCR using primers a and b was carried out for U87-MG cells with overexpressed ATP1A3 or knocked down KLF9. (**N**) Schematic depicting that ATP1A3 stimulation could markedly increase KLF9, which transcriptionally suppresses SNRPG. Error bars = mean ± SD of three experiments and intergroup variabilities were elucidated by the Student t-test. *p < 0.05; **p < 0.01.

Confocal immunofluorescence and Co-IP analyses revealed that in U87-MG cells, ATP1A3 was co-expressed with SNRPG (Figs. **3A** and **3B**). Furthermore, immunoblotting indicated that ATP1A3 overexpression and SNRPG knockdown significantly inhibited the expression of EMT-associated genes in GBM cells (Fig. **3C**). Moreover, SNRGP overexpression significantly reversed the downregulation of EMT-related genes caused by ATP1A3 overexpression (Fig. **3D**). Concluding that SNRPG mediates ATP1A3-induced EMT alterations. To assess how ATP1A3 inhibits SNRPG-induced GBM cells’ malignant growth, the influence of ATP1A3 on SNRPG-mediated EMT in U87-MG cells was assessed. Increased vimentin level suggested that 24 hours of SNRPG exposure induces EMT. ATP1A3-OE cell morphology remained nearly unchanged; however, relatively small changes in EMT markers were observed after SNRPG treatment (Fig. **3E**). A series of protein degradation inhibitors-MG132, leupeptin, and ALLN with ATP1A3, was utilized to treat U87-MG cells. The SNRPG downregulation was similar to that observed with ATP1A3 independently, indicating posttranslational degradation might not be linked with ATP1A3-induced SNRPG downregulation (Fig. **3F**). Indicating that ATP1A3 mediates SNRPG expression reduction, inducing EMT.

The Cistrome database (http://cistrome.org/db/#/) online tool was used to predict the potential transcription factor that regulates SNRPG expression (Fig. **3G**). Then JASPAR (https://jaspar.genereg.net/) online tool predicted two KLF9-binding sites upstream of the SNRPG transcription start site (Fig. **3H**). Subsequently, the cloning sequence comprising these SNRPG promoter region sites was ligated into the pGL3-Basic vector. The constructed fluorescent reporter vector containing the KLF9-binding site was then co-transfected with the overexpression KLF9 vector into GBM cells to confirm that KLF9 binds to the SNRPG promoter region and inhibits its expression. The results have indicated a significant reduction of fluorescence activity in the KLF9-overexpression group (Fig. **3I**), indicating that KLF9 could inhibit SNRPG expression by binding with its promoter. Furthermore, immunoblotting indicated that treatment of recombinant ATP1A3 protein increased KLF9 levels, whereas KLF9 knockdown decreased ATP1A3-induced SNRPG reduction (Fig. **3J**), suggesting that ATP1A3 inhibits SNRPG expression *via* transcriptional interactions. Interestingly, similar results have also been reported in a p53-mutated context using p53-mutated U251-MG cells (Fig. **S2**), and furthermore, the inhibition of ATP1A3 functionality has also been found to exert an effect on SNRPG expression by using ATP1A3 knockdown *via* shRNAs (Fig. **S3**).

As mentioned above, it has been indicated that ATP1A3-induced KLF9 represses SNRPG transcription. ChIP assay was carried out to validate the direct KLF9 binding with the SNRPG promoter. The whole promoter region from -1000 to 200 bp upstream of the transcription start site was surveyed by 4 sets ( ~ 300 bp covered/set) of primers containing 8 pairs. qPCR revealed that the KLF9 was markedly enriched in areas covered by two pairs of primers (from -400 to -100 bp): a and b (Figs. **3K** and **L**). Moreover, KLF9 recruitment was notably increased after the treatment of recombinant ATP1A3 protein and reduced after KLF9 knockdown (Fig. **3M**). The data conclude that ATP1A3 increases the KLF9 expression, which may directly bind to SNRPG and inhibit its transcription and expression (Fig. **3N**).

### 3.4 SNRPG Might Decrease ATP1A3 via Increasing Ser643 Phosphorylation

**Fig. (4):**
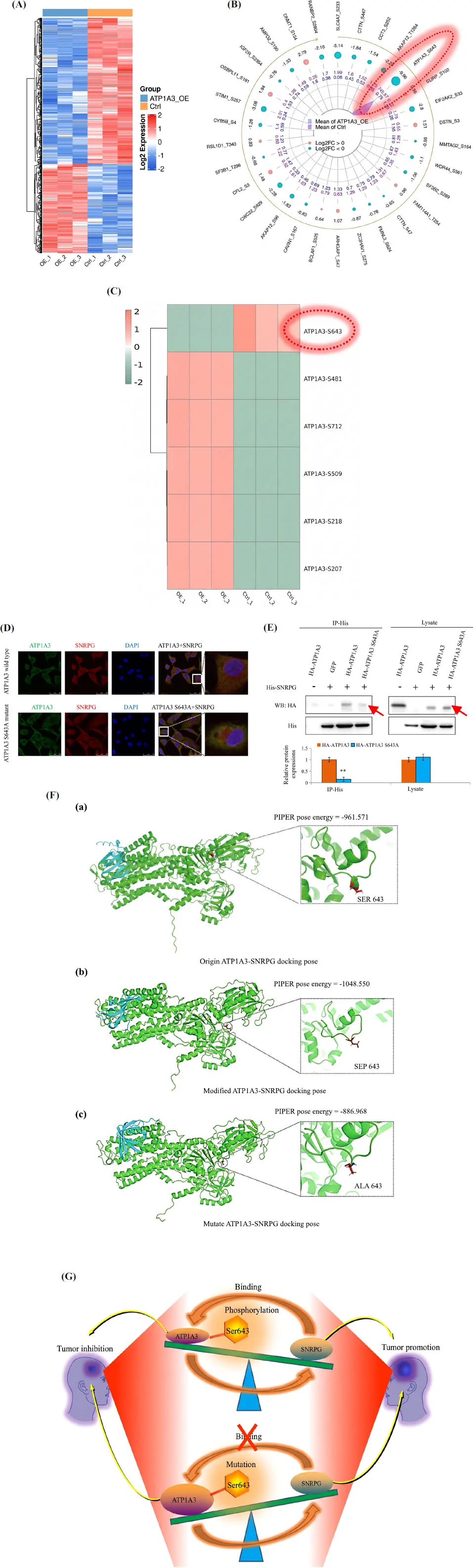
The regulating mechanisms of SNRPG in ATP1A3 Ser643 phosphorylation. (**A**) Intracranial xenograft tissue samples from ATP1A3-OE and control cells were compared, and differential analysis of phosphorylation sites was performed. (**B**, **C**) The Ser643 phosphorylation site indicated a negative correlation with ATP1A3. Analysis of differentially expressed ATP1A3-associated phosphorylation sites. (**D**) Immunofluorescence analysis in U87-MG cells indicated that ATP1A3 S643A mutant was less co-localized with SNRPG. (**E**) ATP1A3 S643A site is required for its interaction with the SNRPG. Red arrowheads indicate decreased protein interactions. (**F**) The predicted binding of SNRPG and ATP1A3 protein with phosphorylation modification (b) and ATP1A3 protein with the Ser643Ala mutation (c) as assessed by Pymol (Version 2.5). The docking score of ATP1A3 phosphorylation modification was −1048.550, while that with Ser643Ala mutation was −886.968, suggesting that the binding strength of mutated ATP1A3 with SNRPG was markedly weak than that of phosphorylated ATP1A3 with SNRPG. (**G**) Suggested mechanisms of the binding regulation between ATP1A3 and SNRPG protein. We suggest that the antagonism between ATP1A3 and SNRPG and regulatory mechanisms could clarify how ATP1A3 regulates GBM progression.

Three ATP1A3-overexpressing GBM and normal control tissue samples were selected for differential analysis of phosphorylation sites (Fig. **4A**, Table **S5**). Significant differences in ATP1A3 Ser643 site expression were detected between ATP1A3-OE and control groups (Fig. **4B**), indicating a significant correlation between ATP1A3 overexpression and reduction of Ser643 phosphorylation (Fig. **4B**). Subsequently, all detected differentially expressed ATP1A3 phosphorylation sites were analyzed, confirming that the Ser643 site was the most prominent one (Fig. **4C**). Since SNRPG inhibits ATP1A3 expression, it was investigated if SNRPG binds to the ATP1A3 Ser643 site. U87-MG cells immunostaining revealed that the Ser643 mutation might reduce the colocalization of ATP1A3 (green) and SNRPG (red) (Fig. **4D**). Immunostaining colocalization was assessed *via* Olympus confocal microscope software. The “Overlap” index ranged from -1 to 1, where 1 = 100% colocalization. The wild-type ATP1A3 localized at endochylema and overlapped with SNRPG (average overlap 0.8621), while S643A mutant ATP1A3 localized at the cell membrane and had reduced overlap with SNRPG (average overlap 0.1301), suggesting the need for Ser643 site phosphorylation for ATP1A3/SNRPG interaction. Moreover, it was assessed if S643 sites were critically linked with ATP1A3-SNRPG interaction, and S643A mutant indicated reduced binding with SNRPG (Fig. **4E**).

Furthermore, it was elucidated if inhibiting the Ser643 amino acid residue interactions influences the binding and activation of ATP1A3 by SNRPG *via* computer simulation. It indicated that certain point mutations and mimic phosphorylation modification influence molecular binding. The information of predicted SNRPG and ATP1A3 binding mode with the mimic phosphorylation modification (b) and ATP1A3 with Ser643Ala mutation (c) are presented in Fig. (**4F**). The ATP1A3 with the mimic phosphorylation modification docking score was −1048.550, and for ATP1A3 with Ser643Ala mutation was −886.968, indicating that the binding between ATP1A3 with mimic phosphorylation and SNRPG was markedly stronger than that between mutated ATP1A3 and SNRPG. These data suggested that point mutation-Ser643Ala could notably interfere with SNRPG and ATP1A3 binding, while ATP1A3 Ser643 phosphorylation modification could stimulate SNRPG-induced ATP1A3 binding and activation (Fig. **4G**). Altogether, deprivation of Ser643 phosphorylation might impact ATP1A3-induced anticancer signaling by blocking the ATP1A3 and SNRPG binding.

### 3.5 The Anti-glioma effects of Synthetic Peptide for Inhibiting ATP1A3 Ser643 Phosphorylation

**Fig. (5):**
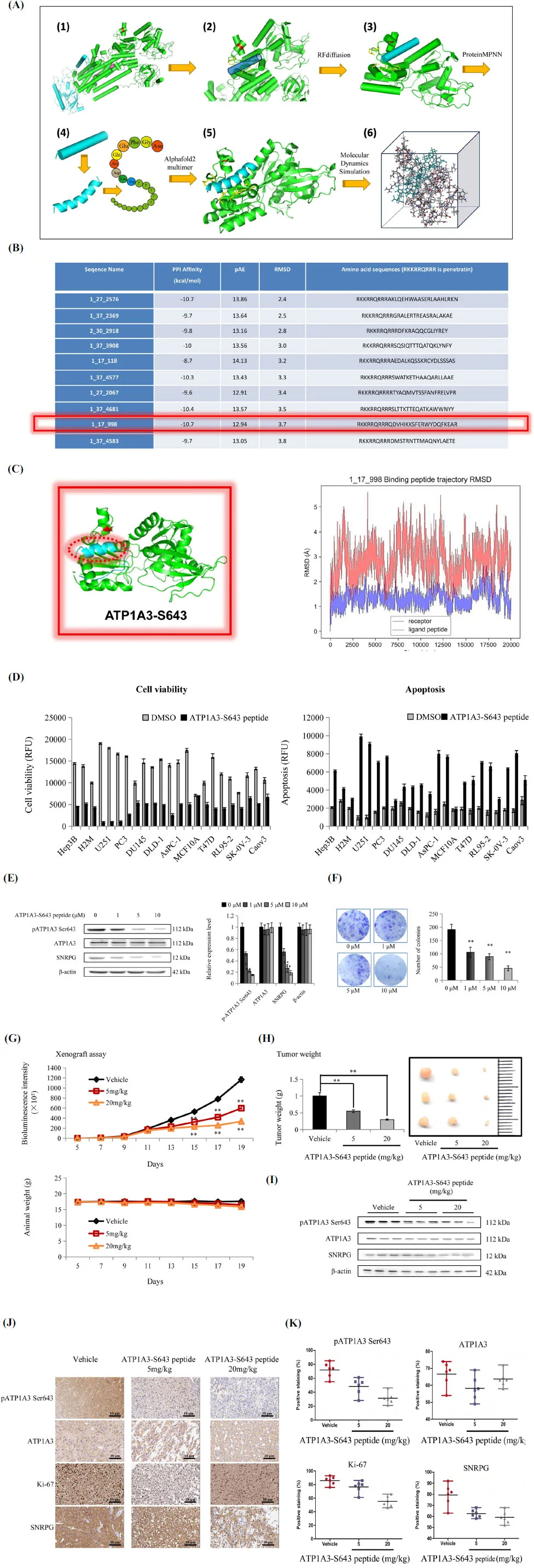
The anti-glioma effects of synthetic peptide for inhibiting ATP1A3 Ser643 phosphorylation. (**A**) Schematic of the *de novo* design procedure. (1) Full-length ATP1A3 crystal structure, derived from Protein Data Bank (PDB ID: 8D3V), the phosphorylase binding site S643 is highlighted in red. (2) Truncated ATP1A3 retained only the residues 349-700. The blue cylinder indicates the potential binding site positioned within the S643 groove. The binder is designed to interact with specific residues from the S643 groove (S643, P647, R648, I655, D659, Q667, and E670), which are shown in yellow. (3) Generate the binder scaffold using the Binder Design module of RFdiffusion, which is indicated in Cyan. (4) Design binder sequences with ProteinMPNN. (5) Validate and rank the folding ability and ATP1A3 binding conformation of designed sequences using Alphafold Multimer. (6) Verify the binding stability by preforming molecular dynamics simulation of the candidate sequences. (**B**) We finally obtained 10 short sequence structures of peptides stably binding to the ATP1A3 S643 phosphorylation site *via* kinetic simulations analysis. Among all these peptides, the most prominent one could be of the highest PPI Affinity score and the lowest pAE score. We name it “ATP1A3-S643 peptide”. (**C**) We then verify the binding stability of the ATP1A3-S643 peptide by preforming molecular dynamics simulation, and we could find that ATP1A3-S643 could be stably bound to the S643 site of ATP1A3. (**D**) ATP1A3-S643 peptide suppresses the activity of various cancer cells and promotes cancer cell apoptosis, including breast, endometrial, ovarian, liver cancer, colon cancer, prostate cancer, and pancreatic cancer cell lines. Left panel shows the results of cell activity measurement, while right panel shows the results of cellular caspase 3/7 activity. (**E**) pATP1A3 Ser643, ATP1A3 and SNRPG protein levels in U87-MG cells were analyzed using Western blotting. All values are denoted as the mean ± SD from ten independent photographs from each group. (**F**) Colony formation in U87-MG tumor cells was also analyzed, and the colony formation rate was calculated. (**G**) Tumor growth kinetics (upper panel) and mice body weights (lower panel), were assessed in different ATP1A3-S643 peptide concentration groups and vehicle groups, ** = *p* ≤ 0.01. (**H**) Tumor weights were assessed in different ATP1A3-S643 peptide concentration groups and vehicle groups, ** = *p* ≤ 0.01). (**I**) Western blotting analysis for the expression of pATP1A3 Ser643, ATP1A3 and SNRPG in the mouse models. (**J**, **K**) IHC analysis of pATP1A3 Ser643, ATP1A3 and SNRPG protein expressions in the tumor samples. All values are denoted as the mean ± SD.

The molecular interactions of ATP1A3 and SNRPG in GBM treatments have been elucidated, as mentioned above. To better explore the effect of the inhibition of ATP1A3 Ser643 phosphorylation on GBM malignancy, seeking potential inhibitors targeting relevant phosphorylation site would be an intriguing research direction. Our previous research has already confirmed the important roles of ATP1A3 in GBM treatment. However, the roles of ATP1A3 phosphorylation still need more research. To further investigate whether the inhibition of ATP1A3 Ser643 phosphorylation could contribute to better prognosis of GBM patients, a synthetic peptide (ATP1A3-S643 peptide) has been designed by AI techniques, which could be the potential inhibitor of ATP1A3 Ser643 phosphorylation (Fig. **5A**). We finally obtained 10 short sequence structures of peptides stably binding to the ATP1A3 S643 phosphorylation site *via* kinetic simulations analysis (Fig. **5B**). Among all these peptides, the most prominent one could be of the highest PPI Affinity score and the lowest pAE score. We name it “ATP1A3-S643 peptide” (Fig. **5C**). We then verified the binding stability of the ATP1A3-S643 peptide by performing a molecular dynamics simulation, and we found that the ATP1A3-S643 peptide was stably bound to the S643 site of ATP1A3. The inhibitory effects of ATP1A3-S643 peptide on cancer cell proliferation, as well as cellular caspase 3/7 activity (apoptosis) of various cancers, have been investigated (Fig. **5D**). *In vitro* research have also revealed that the ATP1A3-S643 peptide-treated groups have markedly decreased expression levels of pATP1A3 Ser643 and SNRPG, while no obvious ATP1A3 alteration could be found (Fig. **5E**). This indicated that the ATP1A3-S643 peptide significantly suppressed the ATP1A3 Ser643 phosphorylation while inhibiting SNRPG expression in U87-MG cells. Consistent with inhibition of cell proliferation, ATP1A3-S643 peptide also significantly inhibited colony formation (Fig. **5F**). Furthermore, *in vivo* research has also been conducted. U87-MG cells were transplanted into BALB/c mice to establish xenografted models and were then given ATP1A3-S643 peptide treatment. It was indicated that ATP1A3-S643 peptide significantly reduced tumor size (Fig. **5G**) and tumor weight (Fig. **5H**) than the vehicle group, and no obvious change in body weight could be found (Fig. **5G**). Besides, *in vivo* research has further the inhibitory effect of ATP1A3-S643 peptide on the expression levels of pATP1A3 Ser643 and SNRPG (Fig. **5I**). IHC has also confirmed the inhibiting effect of ATP1A3-S643 peptide on pATP1A3 Ser643, ATP1A3 and SNRPG expressions (Figs. **5J** and **5K**). Moreover, primary tumors’ Ki-67 staining by IHC also demonstrated that ATP1A3-S643 peptide markedly suppressed the GBM cell proliferation in xenografted mice (Figs. **5H** and **5I**). It could be concluded that the ATP1A3-S643 peptide could reduce the phosphorylation level of ATP1A3 (ser643) *via* binding to the phosphorylation site of ATP1A3 in GBM tissues, subsequently inhibiting cancer cell proliferation more precisely. All these results indicated that ATP1A3 Ser643 phosphorylation inhibitors have great potential for treating GBM.

### 3.6 Bioengineering Nanomedicine Capable of on-demand ATP1A3 Activator Delivery to the Brain for GBM

**Fig. (6):**
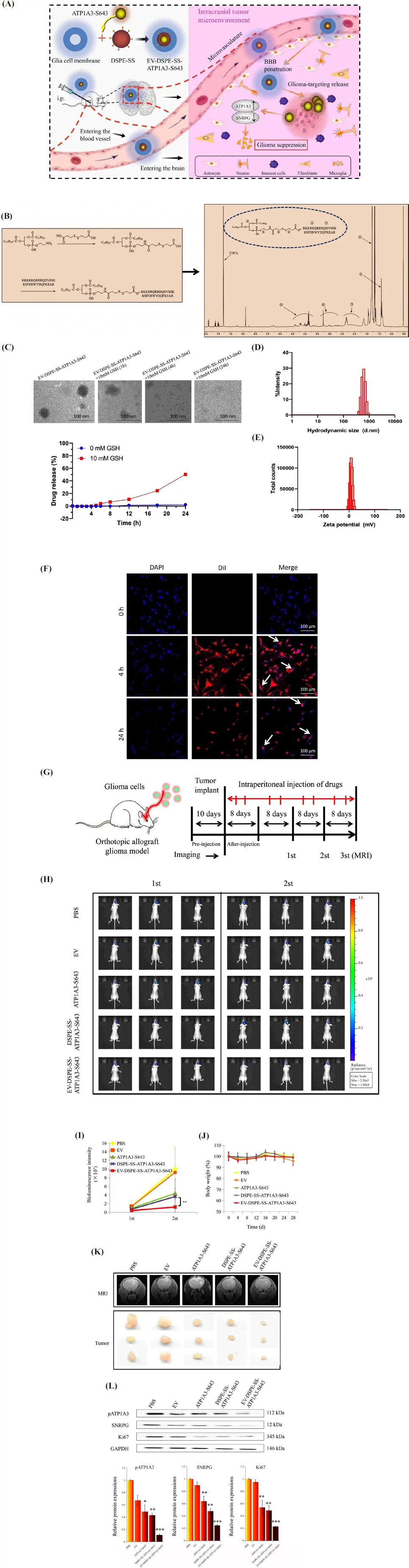
The design and application of engineered nanomedicine to spatiotemporally and sequentially deliver ATP1A3 activator for GBM treatment. (**A**) The illustration of targeted nanodrug system loading with the ATP1A3 activator-ATP1A3-S643, for glioma therapy, as well as its synthesis. (**B**) Molecular construction of DSPE-SS-CS6 and its validation *via* nuclear magnetic resonance (NMR) hydrogen spectrum. (**C**) Detection of drug release *via* electron microscope technique (upper panel) and the drug-release curve of engineered nanomedicine (lower panel). (**D**) The particle size distribution of the engineered nanomedicine is primarily around several hundred nanometers. (**E**) The zeta potential of the engineered nanomedicine is close to electroneutral and could be prone to cell phagocytosis. (**F**) The phagocytosis analysis in U87-MG glioma cells showed that the engineered nanomedicine could enter the tumor cells within 4h, and further release into the nucleus within 24h (see arrow). (**G**) Therapy protocol. (**H**) Bioluminescence imaging of mice treated with PBS, EV, ATP1A3-S643, DSPE-SS-ATP1A3-S643 and EV-DSPE-SS-ATP1A3-S643. (**I**) Quantification of the total flux analyzed by *in vivo* bioluminescence imaging of luciferase activity (n = 3 mice per group). (**J**) The changes of mice body weight during the treatment period were normalized to their initial weight (n = 3, mean ± SD). (**K**) Representative MRI photos of representative mice and corresponding tumors after various treatments (n = 3, mean ± SD). (**L**) Protein expression patterns of ATP1A3, SNRPG, and Ki-67 in different treatment groups (n = 3, mean ± SD). * = *p* ≤ 0.05, ** = *p* ≤ 0.01, *** = *p* ≤ 0.001.

In the current study, we propose an innovative strategy where the extracellular vesicles (EVs) were employed to enhance therapeutic efficacy and prevent tumor growth. The functionalized EVs were used to improve the synergistic therapeutic effect between EVs and drugs (Fig. **6A**), and is expected to boost the treatment efficacy. Specifically, the molecular construction strategy, as well as the validation results *via* nuclear magnetic resonance (NMR) hydrogen spectrum has been presented (Fig. **6B**). Detection of drug release has been exerted by electron microscope technique (Fig. **6C**, upper panel). The results indicated that the nanoparticles were dissociated after adding glutathione (GSH) into the cell culture medium, indicating that the drug could be successfully released. Furthermore, the drug-release curve of this engineered nanomedicine has also been presented (Fig. **6C**, lower panel), and it indicated that in the absence of GSH, the drug was largely not released, and while in the presence of GSH, the engineered EVs-dissociated drug release could be gradually increased after 4 h. Besides, the particle size distribution of engineered nanomedicine is primarily around several hundred nanometers (Fig. **6D**). The material is close to electroneutral and could be prone to cell phagocytosis (Fig. **6E**). Furthermore, by using the laser confocal fluorescence technique, we found that the engineered nanomedicine could enter into GBM cells within 4 h and then be translocated into cell nucleus within 24 h, suggesting that these nanoparticles could be effectively released inside GBM cells (Fig. **6F**).

Tumors were then grown and detected according to therapy protocols (Fig. **6G**). The tumor-bearing mice were randomly divided into 5 groups for treating with (1) PBS, (2) EV, (3) ATP1A3-S643, (4) DSPE-SS-ATP1A3-S643, and (5) EV-DSPE-SS-ATP1A3-S643, respectively, so as to study the treatment effect of the bioengineered EV-coated polymeric nanomedicine. The maximum growth inhibition of the tumor was observed in the therapy group treated by EV-DSPE-SS-ATP1A3-S643 according to the bioluminescence images (Fig. **6H**) and verified by the corresponding semiquantitative bioluminescence analysis (Fig. **6I**), compared with the simple drug delivery and the control groups over the period of the treatment. In addition, the bioengineered EV-coated polymeric nanomedicine only induced slight body weight loss in mice in 28 days, indicating that it was capable of efficiently curbing the recurrence and growth of glioblastoma without adverse effects (Fig. **6J**). After treatments of 28 days, magnetic resonance imaging (MRI) was used to detect the tumor volume (Fig. **6K**). The tumor inhibition rate of ATP1A3-S643, DSPE-SS-ATP1A3-S643, and EV-DSPE-SS-ATP1A3-S643 treatment groups increased significantly compared with PBS and EV groups. The tumor inhibition rate of DSPE-SS-ATP1A3-S643 is higher than that of ATP1A3-S643, and more importantly, the tumor inhibition rate of EV-DSPE-SS-ATP1A3-S643 was the highest than those of other groups. These data indicated that the targeting function of EV-enabled EV-DSPE-SS-ATP1A3-S643 quickly and accurately finds targets and then releases ATP1A3-S643 to inhibit the growth of tumor cells (Fig. **6K**). Besides, the expression patterns of ATP1A3, SNRPG as well as Ki-67 in each group were also examined. It could be found that ATP1A3 expression was more significantly inhibited compared with those of other groups, and the expression level of ATP1A3 appeared negatively correlated with the expression levels of both SNRPG and Ki-67 (Fig. **6L**). All these results indicated that the bioengineering EV-coated polymeric nanomedicine developed in this work exhibited an improved therapeutic efficacy in the ATP1A3-targeted treatment of GBM.

## 4 DISCUSSION

Recent studies have linked increased expression of the Na^+^/K^+^-ATPase on mammalian cell membranes to cancer onset, development, and malignancy [28]. This pump is also crucial in cancer cells as it impacts ion balance, energy metabolism, signal transmission, and morphological structure [29]. Humans have four ATP1 α (ATP1A1-1A4) and three ATP1β (ATP1B1-1B3) isoforms; the expression of these isoforms differs in different tissues. The transmembrane α3 subunit (encoded by *ATP1A3*) is the main functional unit of the Na^+^/K^+^-ATPase [30]. Evidence suggests that ATP1A3 dysfunction could potentially cause various neurological disorders [11] and malignant tumor proliferation, consistent with this research [13]. ATP1A3 functional molecular mechanisms are poorly understood, and knowledge about its cellular targets is limited. Here, it was uncovered that decreased ATP1A3 levels in GBM are related to increased SNRPG, which promotes ATP1A3 S643 phosphorylation and inhibits ATP1A3 expression. However, ATP1A3 overexpression negatively modulates SNRPG-mediated EMT. This investigation is the first to provide evidence of ATP1A3’s crucial role in modulating SNRPG signaling and the antagonism between ATP1A3 and SNRPG in GBM. ATP1A3 suppresses SNRPG-mediated GBM EMT, whereas SNRPG reduces ATP1A3 by increasing Ser643 site phosphorylation. This research also provided new strategies for mechanistic research of other cancers. In the future, new tools and protocols, including Ala-scanning [31], could help determine ATP1A3 active sites to discover other anticancer drugs.

The SNRPG protein is a core element of U1, U2, U4, and U5 snRNP complexes, building blocks of the spliceosome. They might also be U7 snRNP complex components involved in processing the 3’ ends of histone transcripts. Various transcript alternates encoding different SNRPG isoforms were identified (http://www.genecards.org/). SNRPG-linked molecular mechanisms in GBM need clarification [17] as it is a favorable candidate based on gene screening, and our previous research has already confirmed its roles in GBM occurrence, development, and TMZ resistance [17]. Here, the association between SNRPG and ATP1A3 signaling was highly significant for the GBM treatment strategy, which still requires further elucidation. Targeting SNRPG-related signaling could be a potential therapeutic avenue for efficient GBM treatment and overcoming chemoresistance.

Current clinical practice in making individualized treatment decisions continues to depend on the identification of actionable targets. Recently, the advent of novel targeted therapies has positioned targeted treatment as the first-line approach for personalized and comprehensive cancer care [31]. Precision medicine holds great promise in oncology and presents both opportunities and challenges [32, 33]. A major limitation remains the insufficient availability of drugs targeting every potential therapeutic candidate. Developing effective pharmacological designs requires significant time and resources before viable drug candidates can be identified. However, recent advances in machine learning are beginning to show potential in areas such as systematic prediction of compound-target interactions [34], *de novo* design of single-target inhibitors [35], and the identification of existing drugs with potential therapeutic efficacy [36]. Protein modeling has lately seen success using diffusion models. A study by David Baker and others published in Nature shows how RoseTTAFold Diffusion (RFdiffusion) improves the effectiveness of functional protein design [27]. Previous approaches to binder design based solely on target structural information required testing tens of thousands of sequences [37]. However, when RFdiffusion is combined with enhanced filtering methods [38], it results in a two-fold improvement in experimental success rates. This frequently removes the need for time-consuming and expensive high-throughput screening by enabling the identification of high-affinity binders from a small number of designs. The ATP1A3-S643 peptide for inhibiting ATP1A3 Ser643 phosphorylation designed in this study could be a crucial step towards implementing precision medicine for combating GBM. Current research has the potential to play a crucial role in developing targeted therapies for GBM, advancing precision medicine, and fostering the creation of new molecular-targeted drugs.

To improve drug accumulation at tumor locations, nanoparticles are frequently utilized for delivery [39]. The enhanced permeability and retention (EPR) impact supports aberrant blood arteries and impaired lymphatic outflow, promoting nanoparticle accumulation in tumor tissues [40]. As a result, localized nanoparticle drug delivery systems improve drug bioavailability while reducing side effects [41]. However, the EPR effect only facilitates nanoparticle accumulation in tumor tissues, and the limited cellular uptake of the drug delivery system still restricts the effective dosage of anticancer drugs, ultimately diminishing treatment efficacy [41]. Furthermore, the physiological structure of the BBB, consisting of an endothelial cell monolayer surrounded by astrocytes and pericytes [42], significantly impedes the nanoparticle's ability to cross the BBB and reach the tumor. To overcome this challenge, nanoparticles should be conjugated with ligands that target both the BBB and the tumor, thereby enhancing the efficacy of brain tumor drug delivery systems. In the current research, a tumor-targeted nano-drug system (EV-DSPE-SS-ATP1A3-S643) was successfully developed. In this nanomedicine, the EV was used to target the GBM cell, and DSPE was used to bind the EV. DSPE-SS-ATP1A3-S643 could gradually release ATP1A3-S643 under reduction reactions. After entering the tumor cells, GSH could induce the break of a disulfide bond, causing the release of ATP1A3-S643. The level of GSH in tumor cells is 100~1000 times higher than that in the extracellular environment. Thus, GSH is generally considered an ideal intracellular stimulus that could initiate the dissociation of drug carriers and rapid drug release. The EV-DSPE-SS-ATP1A3-S643 group showed a significantly stronger inhibitory effect compared to the other groups, as evidenced by the clear differences in fluorescence intensity. However, future research should include longer treatment durations and focus on the optimization of the therapeutic strategies. Overall, such kind of tumor microenvironment-responsive nanomedicine could effectively reduce the systemic toxicity of the drug and lead to better anti-tumor effects. After that, the *in vivo* study demonstrated the outstanding performance of this bioengineering EV-coated polymeric nanomedicine in GBM treatment without prominent side effects. However, more research should be done to further explore whether the dosage of ATP1A3-S643 could be reduced.

It is widely recognized that mutations in ion channels and transporters are linked with many cancers [43, 44]. The data presented above suggest that variants in these proteins can contribute to the development of malignancies. Its promise as a predictor of patient prognosis and the effectiveness of anticancer treatments is highlighted by this work, the first comprehensive analysis of the functions of ATP1A3 in GBM. Substantial evidence suggests that pathogenic variants in ATP1A3 are potential contributors to various neurological disorders, with multiple studies highlighting the plausible mechanisms through which inactivating mutations of ATP1A3 may lead to these conditions [45]. Therefore, it is crucial to consider that ATP1A3 gene regulation or mutations could give rise to a range of pathologies. Specifically, mutated ATP1A3 may disrupt the regulation of SNRPG or other critical factors involved in cancer progression, warranting further in-depth research. This disruption could also correlate with tumor grade or subtype, as recent studies have indicated that ATP1A3 may influence the levels of EMT-related proteins in GBM cells. Consequently, we believe that the current findings provide essential insights for more extensive systematic investigations of the function of ATP1A3 in GBM cells. This study is important to prioritize large-scale preclinical and clinical studies to investigate further the influence of ATP1A3 alterations on the incidence of various diseases and the clinical characteristics of specific conditions. While ATP1A3 mutations are linked to various CNS disorders [45], the role of ATP1A3 dysfunction in GBM and other cancers requires further studies. The issue of cell line misidentification has been raised in several reports [46], and it is widely acknowledged that the identity of the cell lines used should be verified. Based on STR authentication of the cell line. The results confirmed that the U87-MG cell line used in the current study is suitable for experimental research. Given the substantial circumstantial evidence supporting the origin of this cell line, its use is not considered a reproducibility concern in GBM research. The findings of this study provide an important foundation for future GBM treatment.

Gene therapies are being developed to edit the genome to overcome the therapeutic resistance of GBM [47]. However, despite promising expectations, the inherent heterogeneity of GBM, the wide array of mutations, and immune evasion mechanisms present significant challenges to these approaches and affect the effectiveness of targeting ATP1A3 in clinical settings [48-50]. Additional clinical trials are crucial to establishing standardized protocols and integrating innovative gene strategies into therapeutic synergy. More explorations regarding the feasibility and safety of human patients are urgently needed. However, the proposed mechanisms need to be further confirmed by using more biochemical studies. The current data offer valuable insights for designing more targeted studies, which will be essential for systematically and conclusively assessing the role of ATP1A3 in GBM treatments.

## CONCLUSION

In conclusion, GBM remains one of the most lethal malignancies; new therapies are urgently needed. Evaluating the anti-cancer effects of ATP1A3 may help discover new targets for more efficient therapy. This investigation elucidated the significance of ATP1A3 in GBM. The results suggest that ATP1A3 is a promising GBM treatment target, and the antagonism between ATP1A3 and SNRPG could substantially suppress GBM growth. Besides, a tumor-targeted nano-drug system (EV-DSPE-SS-ATP1A3-S643) of ATP1A3 activator was successfully developed for GBM treatment. These findings strongly support ATP1A3 as a potential target for malignant GBM treatment.

## Data Availability

The datasets used and/or analyzed during the current study are available from the corresponding author on reasonable request. No systematic registered protocol (including the research question, key design features, and analysis plan) was prepared before the study.

## LIST OF ABBREVIATIONS

ATP1A3: Sodium Pump α3 Subunit
BBB: Blood-brain Barrier
GBM: Glioblastoma
IHC: Immunohistochemistry
SNRPG: Small Nuclear Ribonucleoprotein Polypeptide G
TCGA: The Cancer Genome Atlas
